# The Dual-Role of Methylglyoxal in Tumor Progression – Novel Therapeutic Approaches

**DOI:** 10.3389/fonc.2021.645686

**Published:** 2021-03-22

**Authors:** Alessia Leone, Cecilia Nigro, Antonella Nicolò, Immacolata Prevenzano, Pietro Formisano, Francesco Beguinot, Claudia Miele

**Affiliations:** ^1^ URT Genomics of Diabetes, Institute of Experimental Endocrinology and Oncology, National Research Council, Naples, Italy; ^2^ Department of Translational Medicine, Federico II University of Naples, Naples, Italy

**Keywords:** cancer therapy, glycolysis, glyoxalase, methylglyoxal, oncometabolites, tumor progression

## Abstract

One of the hallmarks of cancer cells is their metabolic reprogramming, which includes the preference for the use of anaerobic glycolysis to produce energy, even in presence of normal oxygen levels. This phenomenon, known as “Warburg effect”, leads to the increased production of reactive intermediates. Among these Methylglyoxal (MGO), a reactive dicarbonyl known as the major precursor of the advanced glycated end products (AGEs), is attracting great attention. It has been well established that endogenous MGO levels are increased in several types of cancer, however the MGO contribution in tumor progression is still debated. Although an anti-cancer role was initially attributed to MGO due to its cytotoxicity, emerging evidence has highlighted its pro-tumorigenic role in several types of cancer. These apparently conflicting results are explained by the hormetic potential of MGO, in which lower doses of MGO are able to establish an adaptive response in cancer cells while higher doses cause cellular apoptosis. Therefore, the extent of MGO accumulation and the tumor context are crucial to establish MGO contribution to cancer progression. Several therapeutic approaches have been proposed and are currently under investigation to inhibit the pro-tumorigenic action of MGO. In this review, we provide an overview of the early and latest evidence regarding the role of MGO in cancer, in order to define its contribution in tumor progression, and the therapeutic strategies aimed to counteract the tumor growth.

## Introduction

Cancer represents an important health problem being the leading cause of morbidity and mortality worldwide, with about 18 million new cases and 9.6 million cancer-induced deaths in 2018 (1). There are several risk factors contributing to carcinogenesis; lots of them can be classified as modifiable and include smoke, physical inactivity, obesity and unhealthy diet (2). However, cancer progression depends by changes in cancer cells resulting not only by activating mutations in oncogenes and inactivating mutations in suppressor genes, but also by alterations in cellular metabolism and tumor microenvironment (3).

In the last few years, cancer has emerged as a metabolic disease. In order to survive in adverse conditions, cancer cells develop metabolic adaption which allows their uncontrolled growth and proliferation (4).

One of the main changes in metabolism of cancer cells is represented by their preference for the use of anaerobic glycolysis to produce ATP, regardless of oxygen availability (5). This phenomenon known as “Warburg effect” has been described for the first time by Otto Warburg in the 1920s, when he showed that cultured tumor tissues have a high rate of glucose uptake, lactate secretion and oxygen availability (5). In normal tissue, with oxygen availability, cells use mitochondrial oxidative respiration to produce energy as this process guarantees a higher ATP generation compared to that produced by fermentation of glucose. The reason why cancer cells prefer fermentation of glucose to lactate, even in presence of oxygen-rich conditions and functional mitochondria, is because this process occurs 10-100 time faster than the complete oxidation of glucose in mitochondria (6, 7).

An important consequence of the increased glycolytic flux is the higher production of glycolysis intermediates. Among these, Methylglyoxal (MGO) is a glucose-derived highly reactive dicarbonyl and the major precursor of advanced glycation end-products (AGEs) (8). Compared to glucose, the glucose-derived glycolysis intermediates, especially MGO, form much more glycated proteins in a more rapid way (4). This leads to the AGEs accumulation and the related AGEs-receptor of AGEs (RAGE) pathway activation that contributes to the pathogenesis of many complications in age-related diseases, including cancer, by fostering tissue and cellular dysfunction (9, 10).

## Methylglyoxal Metabolism and Its Mediated Cellular Damage

MGO is a α-oxoaldehyde metabolite, with a molecular weight of 72Da, mainly formed as byproduct of glycolysis starting from the spontaneous degradation of triose phosphate intermediates, glyceraldehyde-3-phosphate (G3P) and dihydroxyacetone phosphate (DHAP) (11). MGO can be produced also by other minor sources such as: i. the degradation of glycated proteins (8), ii. the threonine catabolism in which MGO is produced from aminoacetone oxidation (12) and iii. the ketone body metabolism where hydroxyacetone, derived from acetone hydroxylation, is further oxidized to form MGO (13, 14). MGO production by glycolysis has been estimated to be around 125 µmol/kg cell mass per day (15) and human plasmatic concentrations about 50-150 nM, while intracellular concentration are about 1-4 µM in human cells (16).

MGO action consists in the spontaneous chemical modification of nucleotides, lipids and proteins. It modifies DNA mainly reacting with deoxyguanosine (dG) to form imidazopurinone adduct 3-(2’-deoxyribosyl)-6,7-dihydro-6,7-dihydroxy-6/7-methylimidazo-[2,3-b]purine-9(8)one (MGdG) (17). MGO-derived DNA adducts can result in DNA strand breaks, nucleotide transversions, DNA-DNA crosslinks and DNA-protein crosslinks (4). In the steady state *in vivo*, approximately 9 adducts per 106 nucleotides are produced; anyway, this frequency increases and may be linked to mutagenesis in ageing, diabetes and other disorders, including cancer, characterized by high levels of dicarbonyls (14, 18).

MGO modifies from 1 to 5% of proteins irreversibly interacting with arginine residues to form hydroimidazolone (MGO-H1), the most frequent MGO-derived AGE, argypirimidine and tetrahydropyrimidine (THP). Since arginine residues are most frequently located in the functional sites of proteins (19), glycation in these sites results in protein inactivation and dysfunction (20). In a minor quantity, MGO also interacts with lysine residues to form N_ε_-(1-carboxyethyl) lysine (CEL) and 1,3-di(N_ε_-lysino)-4-methyl-imidazolium (MOLD) (4). Increasing evidence indicates that an accumulation of MGO-modified proteins is associated to several type of cancers (21, 22).

To prevent the MGO harmful effect, mammalian cells have developed some detoxifying enzymatic mechanisms including glyoxalase (Glo), aldoketo reductases (AKRs) and aldehyde dehydrogenases (ALDHs) (23, 24). Among these, Glyoxalases 1 (Glo1) and 2 (Glo2) represent the most important system, committed to the detoxification of the majority of MGO produced. It is present in the cytosolic compartment of all cells (25) and includes: 1. a catalytic amount of reduced glutathione (GSH); 2. the Glo1, acting as the rate-limiting enzyme that catalyzes the conversion of hemithioacetal, formed by non-enzymatic reaction between MGO and GSH, in S-D-lactoylglutathione; 3. the Glo2 that hydrolyzes S-D-lactoylglutathione in D-lactate, thereby reforming GSH (26).

## The Hormetic Role of Methylglyoxal in Cancer

**Figure 1:**
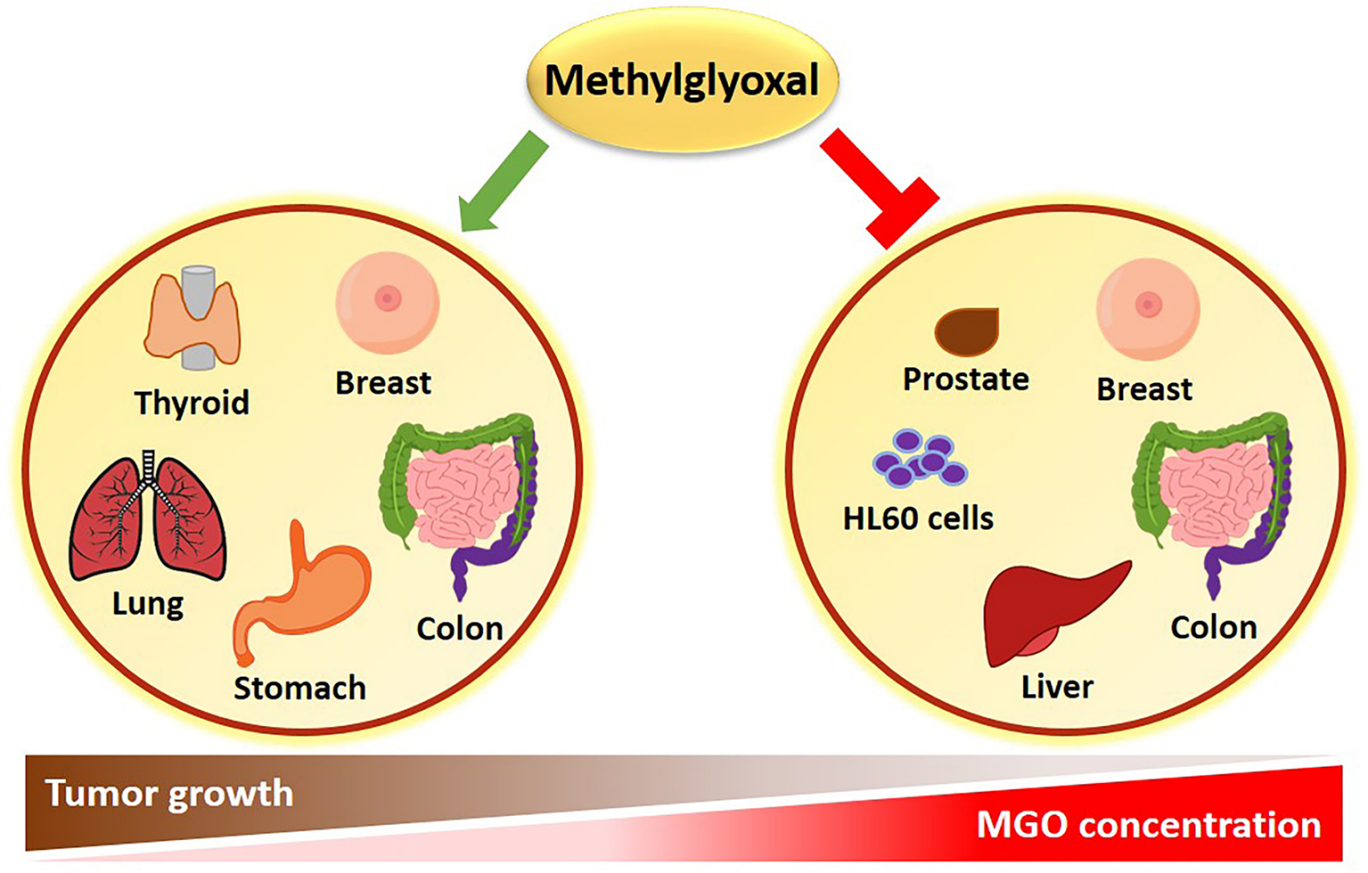
Role of MGO in cancer. An inverse correlation exists between MGO concentrations and tumor progression. High MGO levels cause growth arrest in several types of cancer. Conversely, a lower increase in MGO concentrations can promote cancer growth.

Apparently conflicting data have been published in literature, sustaining both a pro-tumorigenic and an anti-cancer effect of MGO (**Figure 1**). The experimental evidence collected so far, suggests that the dual role of MGO depends on the metabolic adaptation ability of cells. If keeping tolerable, MGO stress results to be beneficial to cancer cells through apoptosis escape and enhanced cell growth. When the threshold of dicarbonyl stress is exceeded, MGO causes major toxic effects on cancer cells (**Figure 1**). This cellular response recalls the hormesis phenomenon, whereby a mild stress-induced stimulation increases cellular stress tolerance and results in beneficial biological effects, whereas cell death represents a final process where failure in adaptation or unhealthy adaptation occurs (27). Molecular effects of MGO on tumor progression are following described and summarized in **Table 1**.

**Table 1:** Effects of MGO and MGO-induced AGEs in different cancer types.

Experimental condition	Model	Cancer type	Effect	Pathways involved	References
**Anti-cancer effects**					
MGO	HL60 EAT	Acute promyelocytic leukemia Ehrlich ascites tumor	Inhibition tumor growth	↓ proliferation ↓ DNA synthesis	(26, 28, 29)
MGO	EAT	Ehrlich ascites tumor	Inhibition tumor growth	↓ mitochondrial respiration ↓ GAPDH activation	(30, 31)
MGO	PC3	prostate	Induction of apoptosis Inhibition of cell cycle Inhibition of glycolytic pathway	Degradation of PARP ↓ cyclinD1, cdk2, cdk4, GAPDH, lactate ↓ RB phosphorylation	(32)
MGO Glo1 inhibitor	MCF7 T47D MDA-MB-231	breast	↓viability, migration, colony formation, tube formation ↑ apoptosis	↑ p-JNK, p-ERK, p-p38 ↓MMP-9, Bcl2	(33)
MGO	DLD-1 SW480 BALB/c nude mice	colon	↓viability, proliferation, migration, invasion ↑ apoptosis	↓ glucose consumption ↓ lactate production ↓ ATP production ↓cMyc expression	(34)
MGO 3-deoxyglucosone	Huh-7 HepG2 Hep3B	liver	↓ migration, invasion, adhesion	↑ p53 nuclear translocation	(35)
**Pro-cancer effects**					
CML Argpyrimidine	NCI-H23 SW1573 Human tissue	lung	Elusion of apoptosis	Hsp27 modification ↓ caspase-3	(36, 37)
MGO	RGK1 HT29	gastrointestinal	↓ apoptosis	Hsp25/Hsp27 modification	(38)
MGO	MDA-MB-231	breast	↑ cell growth ↑ proliferation	Hsp90 modification ↑ nuclear YAP	(39)
Argpyrimidine	Human tissues	colorectal	↑ tumor growth		(40)
MGO	Mouse model	colorectal	↑ glucose levels ↑ LDL/HDL ratio	↑ IL6	(41)
MGO-H1	CAL62 850SC	thyroid	↑ migration, invasion	↑ TGF-β1, p-FAK, MMP1, IL1β	(42)
MGO Glo1 silencing	MDA-MB-231 NOD-SCID mice	breast	↑ metastatic phenotype ↑ ECM remodeling ↑ anchorage-independent growth ability ↑ migration	↑ Tenascin C, CD24, TGF-β1, COL6A1, COL6A2, COL6A3, p-MEK, ERK, SMAD1 ↓ Lumican, COL4A3, COL4A4, DUSP5	(43)
MGO-BSA-AGE	MDA-MB-231	breast	↑ proliferation ↑ migration ↑ survival	↑ MMP9, RAGE, p-ERK1/2, p70S6K1, STAT3, p38	(44)
MGO-BSA-AGE MGO-HSA-AGE	MCF-7	breast	Dose-dependent effect of proliferation, migration, apoptosis	↑ MMP9, p-ERK1/2, CREB1, RAGE ↑ caspase 3 cleavage	(45, 46)

### Methylglyoxal as an Anti-Cancer Metabolite

Pioneering investigations on the biological effects of MGO highlighted an anti-proliferative activity of this Glo substrate (47, 48). Its anti-proliferative activity is characterized by the inhibition of DNA synthesis, protein synthesis and cellular respiration (28). DNA modification by MGO is associated with increased mutation frequency, DNA strand breaks and cytotoxicity, which most likely explain the historically recognized anti-tumor activity of MGO (29–31, 48).

Studies on the mechanisms of MGO toxicity have exhibited marked selectivity for proliferating cells and some selectivity for malignant proliferating cells. Early investigations reported the inhibition of cell growth and toxicity induced by MGO exposure of human leukaemia 60 (HL60) cells *in vitro* (32). The accumulation of nucleic acids and protein adducts preceded the cellular apoptosis in these cells (33). Conversely, a no significant inhibition of cell growth was found in mature peripheral leucocytes (neutrophils and lymphocytes), demonstrating a selectivity of MGO toxicity for HL60 cells with a higher rate of cell growth than mature leucocytes (32, 33). Similarly, MGO treatment inhibited mitochondrial respiration of several types of malignant cells and tissues but it had no inhibitory effect on the respiration of any of the normal cells and tissues tested (34). In Ehrlich ascites cells, this tumoricidal effect of MGO was attributed to the potent inactivation of glyceraldehyde-3-phosphate dehydrogenase (GAPDH), which plays an important role in the high glycolytic capacity of the malignant cells (35).

Millimolar concentration of MGO induces apoptosis in various cancer cell types. Apoptosis was primary due to the block of cell cycle progression and glycolytic pathway in prostate cancer (PC3) cells (49). The activation of mitogen-activated protein kinase (MAPK) family (p-JNK, p-ERK and p-p38 levels) and the downregulation of B-cell lymphoma 2 (Bcl-2) and matrix metalloproteinase 9 (MMP-9) were demonstrated to impair cell viability, proliferation, migration, invasion, tubule formation and increase apoptosis in breast cancer cells (50). These effects were also associated to the impairment of c-Myc expression and glycolytic metabolism in human colon cancer cell lines (51). A study performed in liver cancer cells reported that much lower concentrations of MGO (1 μM) were able to decrease migration, invasion and adhesion of liver cancer cells, without impairing cell viability, in a p53-dependent manner (52). This implies that MGO could find a clinical benefit only in case of metastatic p53-expressing liver cancer. However, these preliminary data were obtained *in vitro* in cancer cell lines without considering the impact on the tumor microenvironment or the systemic effects.

The anti-tumor activity of MGO was also reported *in vivo* in rodents inoculated with tumor cells. The tumor growth was inhibited by single or continuous MGO administration (29–31, 51, 53, 54). However, the potential efficacy of MGO as anti-cancer therapy was limited by the evidence of tumor regrowth once therapy was terminated. The small increase observed in median survival time may be due to a generalized MGO toxicity because of the use of doses close to the maximum tolerated dose (48). An open issue is on the side-effect of MGO in the context of chronic inflammatory conditions, like obesity and diabetes, which represent common risk factors for many tumors, e.g. hepatocellular carcinoma (HCC) and breast cancer.

### Glyoxalase 1 Expression: A Survival Mechanism of Cancer Cells

To compensate for high MGO levels, cancer cells may adopt survival mechanisms including Glo1 increased expression and activity. Indeed, higher levels of Glo1 have been described in several cancers (55–57) and have also been linked to multidrug resistance (MDR) in cancer chemotherapy (55).

The pro-tumorigenic role, initially attributed to Glo1, has been further validated in the last decade by studies sustaining its role as potential target in cancer therapies. The overexpression of Glo1 has been found in biopsies of pancreatic cancerous tissue (58) and HCC (59) but not in the adjacent non-cancerous tissue. In tumor cells of oropharyngeal squamous cell carcinoma (OPSCC), Glo1 expression is positively correlated to argypirimidine modification, and Glo1 protein levels are increased following exogenous MGO administration (60). This suggests that Glo1 expression represents an adaptive response to the accumulation of cytotoxic metabolites and an independent risk factor for unfavorable prognosis of OPSCC patients (60).

Amplification of Glo1 gene has been found to be a frequent genetic event in breast cancer, sarcoma, non-small-cell lung cancer (NSCLC) and, more recently, in HCC (61, 62). Interestingly, xenograft tumor growth is inhibited when Glo1 is silenced in HCC cells carrying genetic amplification but not in cells with normal copies (62), supporting the potential use of Glo1 as target in tailored therapies in patients with genetic Glo1 amplification.

In many tumors, it has been reported that the expression of Glo1 is higher in more aggressive and invasive cells than in less aggressive tumor cells, as described in: PC3 and LNCaP cell lines of prostate cancer, MDA and MCF-7 cell lines of breast cancer, skin carcinomas and skin benign neoplasms, respectively (63–65). Moreover, increased circulating levels of Glo1 have also been found in patients with metastatic compared to non-metastatic prostate cancer (66). These evidence suggest a prognostic role for Glo1 tumor expression (36), as further indicated in fibrosarcoma progression by a proteomic analysis (38). Furthermore, Glo1 emerged as an independent prognosticator of adverse significance in a colorectal cancer (CRC) patient cohort (37), later confirmed by the study showing that patients with low Glo1-expressing CRC had longer disease-free survival than the patients whose tumor expressed higher levels of Glo1 (39).

Some of these studies have described the pro-survival effect of Glo1 as a result of apoptosis elusion, rather than a direct regulation of cell proliferation (59, 62–64). In human CRC cells, Glo1 silencing inhibits colony formation, migration, invasion and induces apoptosis through the increase of the signal transducer and activator of transcription (STAT) 1, p53 and Bax and the decrease of c-Myc and Bcl-2 expression (39). The same pathways have been described in the apoptosis induced in tumor cells by high MGO levels (50–52), which are likely at least part of the effect obtained by Glo1 interfering.

The antitumor effect of Glo1 depletion underlines the potential role of Glo1 as therapeutic target. Beside the prognostic role described above for Glo1, its differential expression in malignant and less aggressive tumor cells may be useful for differential diagnosis.

### Methylglyoxal as a Pro-Cancer Metabolite

Besides the anti-tumor activity, in the last few years more and more evidence have shed light on the MGO ability to promote tumor progression (4).

Endogenous MGO-modified heat shock protein (Hsp) 27 has been found in several types of human cancer, including non-small cell lung (40) and gastrointestinal (41) cancer, where MGO protects cancer cells from apoptosis by increasing the anti-apoptotic activity of Hsp27 through the inhibition of caspase-3 and 9 activation (40–42). In breast cancer cells, MGO-induced post-translational glycation of Hsp90 affects its activity with a consequent decrease of the large tumor suppressor 1 (LATS1) expression, a key kinase for the regulation of the Hippo tumor suppressor pathway through Yes-associated protein (YAP) (43). This study demonstrated that, following MGO accumulation, YAP is retained in the nucleus where it promotes cell growth and proliferation by inducing the expression of genes involved in these processes (43).

More recently, novel molecular mechanisms related to the pro-oncogenic activity of MGO have been identified in different cancer tissues and cell lines.

In CRC human tissues, accumulation of MGO adducts (argypirimidine) are found to be positively correlated with primary tumor staging, indicating that the degree of dicarbonyl-induced stress is associated with CRC tumor aggressiveness (44). Consistent with this data, in experimental mouse models of colon cancer, MGO administration (50 mg/kg BW) causes low-grade carbonyl stress that can lead to inflammation and oxidative stress, responsible for chemically-induced colonic preneoplastic lesions deterioration. Moreover, MGO induces the growth of mouse CT26 colon cancer isografts by enhancing the expression or activation of proteins involved in cell survival, proliferation, migration and invasion (45).

*In vitro* experiments accomplished in anaplastic thyroid cancer (ATC) cell lines showed that MGO-H1 accumulation causes the increase of invasion/migration properties and a marked mesenchymal phenotype through a novel mechanism involving transforming growth factor β 1 (TGF-β1)/focal adhesion kinase (FAK) signaling (46). Similar effects are found in Glo1-depleted breast cancer cells where, the downregulation of the dual specificity phosphate 5 (DUSP5) phosphatase and the consequent over-activation of MEK/ERK/SMAD1 pathway promote the establishment of a metastatic phenotype characterized by increased cell migration and extracellular matrix (ECM) remodeling (67).

Treatment of estrogen receptor (ER)-negative MDA-MB-231 breast cancer cell line with different concentrations of MGO-derived bovine serum albumin AGEs (MGO-BSA-AGEs) causes an increase of the proliferation, migration and invasion capacity in a RAGE-dependent manner. This effect is mediated by a higher MMP-9 activity and phosphorylation of several proteins, including the ribosomal protein serine S6 kinase (p70S6K)-beta 1, STAT3, the p38 MAPK, the glycogen synthase-serine kinase (GSK)-3α and the MAPK/ERK protein-serine kinase 1/2 (MKK1/2), each of these belonging to the main signal pathways involved in tumor growth (68). Similar effects are also induced, in a dose-dependent way, in ER-positive MCF-7 human breast cancer cell line (69). In detail, MCF-7 treated with low doses (50-100 µg/ml) of MGO-BSA-AGEs show a significantly increase of cell proliferation and migration, without any alteration of cell invasion, due to the MAPK pathway activation and cAMP-response element binding protein (CREB1) 1 phosphorylation. Conversely, higher dose (200 µg/ml) of MGO-BSA-AGEs results in cytotoxicity through the activation of the apoptosis mediated by the caspase-3 cleavage (69). Probably, the inhibitory mitogenic effect observed following treatment with 200 µg/ml MGO-BSA-AGEs is due to an impediment to the correct RAGE oligomerization and consequent downstream pathways activation (69). Consistently, Khan M.S. et al. demonstrated that treatment of MCF-7 with MGO-derived human serum albumin AGEs (MGO-HSA-AGEs) (50 µg/ml) causes an increase in cell migration, likely through rearrangements of cytoskeleton (70).

These studies indicate that the conflicting literature available on the role of MGO in cancer progression could be explained by its “hormetic effect”, by which exposure to low doses of MGO causes an adaptive effect crucial for cancer cell survival. Indeed, following MGO treatment, cancer cells show a characteristic biphasic dose response growth curve (71). Moreover, difference in tumor cells response may depend on different ability of cells to face a stress condition. An increased formation of MGO in tumor cells that is not followed by a parallel increase in Glo1 activity or other detoxifying mechanisms results in toxicity (72). Thus, the correct balance between Glo1 activity and the increased MGO production, associated to the high glycolytic flux in cancer cells, is likely crucial for tumor growth response (72).

## Methylglyoxal and Its Derived Adducts as Novel Biomarkers

A promising field of research is the use of MGO or MGO-derived adducts as novel biomarkers for cancer. In support of this, serum carboxymethyl lysine (CML) content has been detected at higher levels in a mouse model of lung cancer compared to controls (73). Consistently, a recent study by Irigaray and Belpomme has proved that free MGO blood levels increase in rats following subcutaneous administration of a tumorigenic cell clone of colon adenocarcinoma, compared to those measured in rats receiving non-growing tumor-associated clone. In addition, the increase of MGO levels correlates with the growth of implanted tumorigenic cell clone, indicating that MGO levels could also represent a useful marker in monitoring tumor progression (74).

Besides proteins, MGO modifies DNA thus enhancing its antigenicity. It has been demonstrated that, circulating autoantibodies against MGO-modified DNA are detectable in cancer patients and their use as markers could be relevant in certain types of cancer (75). Interestingly, a highly sensitive method has been later developed and validated for the simultaneous quantitation of 9 exocyclic DNA adducts derived from 8 aldehydes, including MGO, in human blood samples (76).

According to these data, in a pioneering study, Coluccio et al. have recently demonstrated a positive correlation between MGO-derived adducts and cancer staging by analyzing the secretome of blood-derived circulating cancer cells (BDCs) isolated from blood samples of cancer and healthy patients. This study provides a non-invasive method to detect dynamic changes of cancer in real time that may be used for alternative and personalized pharmacological strategies (77).

Together these studies spotlight the potential for a prognostic role of MGO and its derived adducts in cancer. Further clinical trials will be necessary to benefit from these early evidence and obtain a diagnostic application for MGO-derived adducts detection.

## Therapeutic Strategies

**Figure 2:**
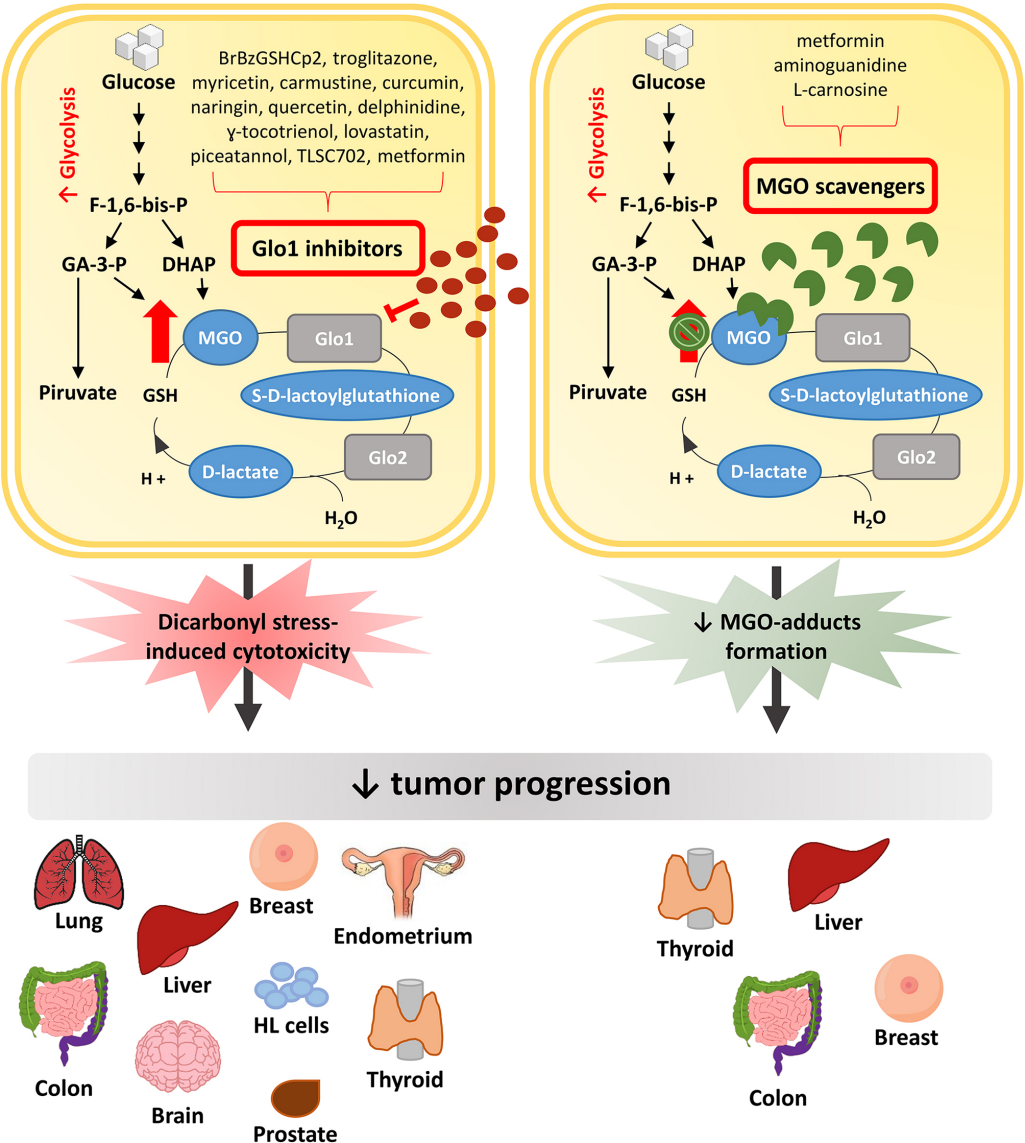
Anti-tumor pharmacological strategies. In cancer cells, the increased glycolytic flux causes a higher MGO production which can sustain tumor growth. Glo1 inhibitors block tumor progression by further increasing MGO intracellular levels and leading to dicarbonyl stress-induced cytotoxicity. Differently, MGO scavengers, by trapping MGO and reducing MGO-induced adducts formation, are able to block tumor progression preventing the cytotoxicity related to high MGO levels. MGO, methylglyoxal; Glo1, Glyoxalase 1; Glo2, Glyoxalase 2; F-1,6-bis-p, fructose-1,6-bis-phosphate; GA-3-P, glyceraldehyde 3-phosphate; DHAP, dihydroxyacetone phosphate; HL cells, Human Leukemia cells.

In this context, major efforts have been made to discover pharmacological approaches able to inhibit, or at least slow down, tumor growth (**Figure 2**). Possible strategies to this purpose have been initially focused on exploiting the cytotoxic effect of high MGO levels (9). Encouraging anti-cancer effects of a MGO-based formulation were early described in pre-clinical models and in a three-phase clinical study (78). Metronomic doses of MGO are able to sensitize breast cancer cells to doxorubicin and cisplatin, thus inducing cell death, without any additional deleterious effects (79). Given the association between Glo1 overexpression and the MDR in cancer chemotherapy, and the presence of Glo1 amplification in several human tumors, an anticancer strategy is further represented by the use of Glo1 inhibitory drugs (56, 80).

The GSH conjugate S-p-bromobenzylglutathione cyclopentyl diester (BrBzGSHCp2) exhibits a Glo1 inhibitor activity (81). The use of BrBzGSHCp2 has been proven to be effective both *in vitro* and *in vivo* models where Glo1 is expressed at high levels, as demonstrated in lung cancer cell line and mouse model (82), in drug resistant leukemia cells (83) and in Huh7 HCC cell line (84). Moreover, combination of BrBzGSHCp2 with sorafenib enhances susceptibility of HCC to the latter (84). Conversely, BrBzGSHCp2 treatment has been associated to a strengthening of aggressiveness in ATC and breast cancer (43, 46). This suggests that the efficacy of anti-tumor strategies involving the use of BrBzGSHCp2 can be ascribed to differences in cell lines and animal models used and, in general, is more efficient in a neoplastic context where Glo1 is expressed at high levels (4). Besides BrBzGSHCp2, troglitazone reduces the Glo1-induced MDR in doxorubicin-resistant K562 leukemia cells, in doxorubicin-resistant MCF7 cells and in astrocytoma cell line U-373 (85–87).

Several compounds are emerging as Glo1 inhibitor, namely curcumin, naringin, carmustine, myricetin, quercetin, delphinidine, γ-tocotrienol, lovastatin, piceatannol and TLSC702. Among these, curcumin is a polyphenol with anti-oxidant, antibacterial, anti-inflammatory, antidiabetic and anti-tumor activities (4, 88). It is known to hamper breast cancer, prostate cancer and brain astrocytoma cell growth (89, 90). By inhibiting Glo1 activity, naringin and carmustine induce apoptosis of human colon adenocarcinoma (Caco-2) cells and PC3 cells (91–93). While myricetin, quercetin, delphinidine, γ-tocotrienol, and lovastatin have been demonstrated to induce apoptosis in HL60 cells (94–98). Through their Glo1 inhibitor activity TLSC702 and piceatannol, a naturally occurring stilbene, reduce the proliferation of human non-small cell lung cancer cells expressing high Glo1 levels (99–101).

Following Glo1 inhibition, cancer cells switch from glycolysis to tricarboxylic acid (TCA) cycle to avoid apoptosis induced by MGO accumulation. Shimada et al. reported that the combination of TLSC702 with shikonin, a specific inhibitor of pyruvate kinase M2 that is a driver of TCA cycle, suppressed the metabolic shift from glycolysis to mitochondrial respiration (TCA cycle), leading to apoptosis of human non-small cell lung cancer (NCI-H522) cells (102). Moreover, TLSC702 decreases cell viability and suppresses tumor-sphere formation in ALDH1-positive cancer stem cells (CSCs) in breast cancer (103).

Metformin (N,N-dimethylbiguanide), a potent anti-diabetic molecule also used in cancer treatment for its anti-tumorigenic properties (104, 105), sensitizes endometrial cancer to progestin by targeting Tet methylcytosine dioxygenase 1 (TET1), which downregulates Glo1 expression (106). Through the inhibition of Glo1 expression, metformin overcomes resistance to chemotherapy. Indeed, combined treatment of metformin with chemotherapeutic drugs, such as cisplatin and paclitaxel, reverses progestin resistance and enhances the sensitivity of endometrial cancer cells to chemotherapeutic drugs (104, 107). Moreover, Antognelli et al. demonstrated that metformin inhibits Glo1 thus hampering epithelial to mesenchymal transition (EMT), migration and invasion of metastatic PC3 cells (66). Besides its effect on Glo1 expression, metformin is emerging as MGO scavenger (108), anyway the knowledge about its action in this context is still limited.

A second and more promising anti-cancer strategy is represented by the use of MGO scavengers, in light of the pro-oncogenic effects of low doses of MGO. Indeed, the anti-cancer strategy based on Glo1 inhibition may result in potential toxic side effects related to the increasing MGO concentration, thus limiting their use in clinical practice.

Aminoguanidine (AG), a diamine oxidase with inhibitor activity on inducible nitric oxide synthase (iNOS), is a well known AGE inhibitor and MGO scavenger (9, 109). First evidence of the anti-cancer effect of AG were provided in thyroid follicular carcinoma, HCC and breast cancer progression, where AG had inhibitory effect on tumor growth by modulating iNOS (110–112). Anyway, there was no link between the AG anti-glycation action and its anticancer effect. Recently, the action of AG on the reversion of MGO pro-cancer effect has been demonstrated in breast cancer cells (67). Similarly, Antognelli et al. have demonstrated that AG treatment in ATC cell line is able to revert the pro-tumorigenic role of MGO and this effect is enhanced when AG is used in combination with resveratrol, a Glo1 activator (46).

L-Carnosine (β-alanyl-L-histidine), a naturally occurring dipeptide acting as MGO scavenger, has been shown to exert anti-proliferative effects in cancer cells (113, 114). It reverses MGO pro-tumorigenic action by decreasing migratory ability of breast cancer cells (43, 67) and inducing apoptosis in CRC cells, both *in vitro* and *in vivo* (44). Moreover, Bellier et al. demonstrated that the combined use of carnosine with cetuximab increases the apoptosis of KRAS-mutated CRC cells, unlike cetuximab treatment alone that has no effect. This effect has been also confirmed *in vivo* in mouse models (115).

Combination of natural compounds with chemotherapeutic drugs is attracting great attention for their low toxicity and potential efficacy against resistant tumors.

## Conclusions

The increasing number of cancer patients worldwide makes the search in this field more challenging. A hallmark of cancer is the altered cellular metabolism that leads to changes in the reactive metabolic intermediates levels, also called “oncometabolites”, which influence cancer progression (116). The preferential use of anaerobic glycolysis implicates a higher formation of endogenous metabolites, such as MGO, in the highly proliferative and metabolically active cancer cells.

MGO contribution to tumor progression is still a debated topic, considering the apparently contrasting literature that attributes to this dicarbonyl both a pro- and anti-cancer effect. Indeed, the studies collected in this review indicate that, on one hand, MGO inhibits tumor growth by inducing cytotoxicity and impairing the expression or activity of factors having a pivotal role in invasiveness. On the other hand, recent studies demonstrate that MGO can support tumor growth essentially through the evasion from programmed cell death and the increased migration, invasion and ECM remodeling processes. This opposite action of MGO can be explained by several factors. First, the extent of MGO accumulation is essential to decide cancer cell destiny. Low levels of MGO are able to promote tumor growth as result of stress-responsive activation of survival mechanisms and apoptosis elusion, while high MGO accumulation induces cytotoxicity. Secondly, the different ability of cancer cells to face MGO-induced dicarbonyl stress is crucial for their survival. Indeed, overexpression of Glo1 is often present in more aggressive tumors and it is associated to MDR.

Many efforts have been made in the search of pharmacological strategies able to exploit the MGO cytotoxic action. Anyway, a promising strategy is represented by the use of natural compounds that can be used in association of chemotherapeutic drugs, with the advantage of showing minor risk of toxic side effects.

Given the complexity of carcinogenesis, further studies are needed to clarify the molecular pathways affected by MGO and involved in tumor progression. This will allow to identify and to optimize therapeutic strategies for personalized treatment of different types of cancer.

## Author Contributions

AL, CN and CM conceived the idea and edited the manuscript. AL, CN, AN and IP wrote the paper. AL and CN prepared the figures. FB, PF and CM reviewed the manuscript. All authors contributed to the article and approved the submitted version.

## Funding

This study was funded, in part, by the Ministero dell’Istruzione, Università e della Ricerca Scientifica (grants PRIN 2017 and PON “RICERCA E INNOVAZIONE” 2014 - 2020 E FSC - progetto “Innovative Devices For SHAping the RIsk of Diabetes” (IDF SHARID) -ARS01_01270), by the Regione Campania (POR FESR 2014-2020 – Obiettivo specifico 1.2. - “Manifestazione di Interesse per la Realizzazione di Technology Platform nell’ambito della Lotta alle Patologie Oncologiche” - Projects COEPICA, RARE PLAT NET and SATIN), and by the European Foundation for the Study of Diabetes (EFSD)/Boehringer Ingelheim (2018-2020). The funders were not involved in the study design, collection, analysis, interpretation of data, the writing of this article or the decision to submit it for publication.

## Conflict of Interest

The authors declare that the research was conducted in the absence of any commercial or financial relationships that could be construed as a potential conflict of interest.

## Abbreviations

AG, Aminoguanidine; AGEs, Advanced glycation end-products; ALDH, Aldehyde dehydrogenase 1; ATC, Anaplastic thyroid cancer; Bcl-2, B-cell lymphoma 2; BrBzGSHCp2, S-p-bromobenzylglutathione cyclopentyl diester; CRC, Colorectal cancer; ECM, Extracellular matrix; ER, Estrogen receptor; Glo1, Glyoxalase 1; GSH, Glutathione; HCC, Hepatocellular carcinoma; HL60, Leukaemia 60 cells; HSP, Heat shock proteins; iNOS, Inducible nitric oxide synthase; MAPK, Mitogen-activated protein kinase; MDR, Multidrug resistance; MGO-H1, Hydroimidazolone; MGO, Methylglyoxal; MGO-BSA-AGEs, MGO-derived bovine serum albumin AGEs; MMP-9, Matrix metalloproteinase 9; OPSCC, Oropharyngeal squamous cell carcinoma; PC3, Prostate cancer cells; RAGE, Receptor for advanced glycation end products; STAT, Signal transducer and activator of transcription; TCA, Tricarboxylic acid cycle; YAP, Yes-associated protein.
